# Intensity-dependent cardiopulmonary response during and after strength training

**DOI:** 10.1038/s41598-023-33873-x

**Published:** 2023-04-24

**Authors:** Johannes Lässing, Tom Maudrich, Rouven Kenville, Zarah Uyar, Christian Bischoff, Sven Fikenzer, Martin Busse, Roberto Falz

**Affiliations:** 1 Department of Exercise Science and Sports Medicine, Martin Luther University Halle-Wittenberg, Von-Seckendorff-Platz 2, 06120 Halle (Saale), Germany; 2 Institute of Sport Medicine and Prevention, University of Leipzig, Rosa-Luxemburg-Straße 20-30, 04103 Leipzig, Germany; 3 Department of Movement Neuroscience, Faculty of Sports Science, Leipzig University, 04109 Leipzig, Germany; 4 Klinik und Poliklinik für Kardiologie, Universitätsklinikum Leipzig, Liebigstr. 20, 04103 Leipzig, Germany

**Keywords:** Cardiovascular biology, Circulation, Respiration

## Abstract

Whereas cardiopulmonary responses are well understood in endurance training, they are rarely described in strength training. This cross-over study examined acute cardiopulmonary responses in strength training. Fourteen healthy male strength training-experienced participants (age 24.5 ± 2.9 years; BMI 24.1 ± 2.0 kg/m^2^) were randomly assigned into three strength training sessions (three sets of ten repetitions) with different intensities (50%, 62,5%, and 75% of the 3-Repetition Maximum) of squats in a smith machine. Cardiopulmonary (impedance cardiography, ergo-spirometry) responses were continuously monitored. During exercise period, heart rate (HR 143 ± 16 vs. 132 ± 15 vs. 129 ± 18 bpm, respectively; p < 0.01; η^2^_p_ 0.54) and cardiac output (CO: 16.7 ± 3.7 vs. 14.3 ± 2.5 vs. 13.6 ± 2.4 l/min, respectively; p < 0.01; η^2^_p_ 0.56) were higher at 75% of 3-RM compared to those at the other intensities. We noted similar stroke volume (SV: p = 0.08; η^2^_p_ 0.18) and end-diastolic volume (EDV: p = 0.49). Ventilation (V_E_) was higher at 75% compared to 62.5% and 50% (44.0 ± 8.0 vs. 39.6 ± 10.4 vs. 37.6 ± 7.7 l/min, respectively; p < 0.01; η^2^_p_ 0.56). Respiration rate (RR; p = .16; η^2^_p_ 0.13), tidal volume (VT: p = 0.41; η^2^_p_ 0.07) and oxygen uptake (VO_2_: p = 0.11; η^2^_p_ 0.16) did not differ between intensities. High systolic and diastolic blood pressure were evident (62.5% 3-RM 197 ± 22.4/108.8 ± 13.4 mmHG). During the post-exercise period (60 s), SV, CO, V_E_, VO_2,_ and VCO_2_ were higher (p < 0.01) than during the exercise period, and the pulmonary parameters differed markedly between intensities (V_E_ p < 0.01; RR p < 0.01; VT p = 0.02; VO_2_ p < 0.01; VCO_2_ p < 0.01). Despite the differences in strength training intensity, the cardiopulmonary response reveals significant differences predominantly during the post-exercise period. Intensity-induced breath holding induces high blood pressure peaks and cardiopulmonary recovery effects after exercise.

## Introduction

The preventive and rehabilitative effects of physical exercise have generally been well-studied^1–6^. Strength training (ST) is an essential part of the guidelines for physical activity for health maintenance and for chronic diseases or disabilities^7^.

The cellular response to ST is widely acknowledged, as are the associated hypertrophic effects of skeletal muscle^8–10^. With regard to morphological adaptations of the cardiac muscle, effects of ST are described as concentric hypertrophy (wall thickening), whereas endurance training induces eccentric hypertrophy (the internal dimensions increase, as does the thickness of the wall)^11,12^.

Not conclusively clarified and unlike endurance training, the acute hemodynamic adaptations (stroke volume and cardiac output) of strength exercises have rarely been described^13^. The stroke volume und cardiac output response during body-weight strength training (5 body-weight exercises; e.g. squats, push-ups, inverted rows, isometric exercises) seems lower than during endurance training (continuous cycling; 70% heart rate max)^14^. Resistance (heavy weight-lifting or isometric exercise) training-induced blood pressure increase, on the other hand, is extrem elevated at high intensities^15,16^. There is evidence that executing strength training (e.g. high load/low repetition or low load/high repetition) has a significant influence on the amount of cardiovascular exertion during and after exercise^17,18^. Moreover, the amount of exercised muscle mass (comparing different strength exercises) has an impact on cardiovascular response in strength training^19^.

However, strength training protocols differ with respect to working-muscle groups, modality (dynamic vs. isometric, body weight vs. device-supported), duration, and intensity. Whereby, the intensity and cumulative volume of strength training protocols exert a decisive influence on muscular adaptations^20–22^.

Although strength training has grown increasingly popular in recent decades and regular strength training’s long-term effects have been widely documented^23–27^, the acute cardiopulmonary response moreover is often only investigated by relying on standard parameters like heart rate and oxygen uptake. Hemodynamic parameters have been primarily analyzed in conjunction with endurance training^28,29^ and seldom in strength training, whereby the few studies differ regarding the comparison groups in intensity, number of repetitions^17,18^, or used muscle mass^19^. Gjovaag et al. compared high load/low repetition resistance training (RT) to low load/high repetition RT and found significant different blood pressure and cardiac output responses during and after exercise. Also, during different strength exercises, the involved muscle mass showed different hemodynamic responses^19^. However, to date, there has been no investigation comparing the acute hemodynamic response in standardized strength training with the same number of repetitions at different intensities. In addition, breath holding is a natural reflex triggered during resistance exercises when greater effort is required^15,30^, which is likely to affect cardiac parameters. Therefore, dose–response relationships between strength training intensity and cardiopulmonary effort are important for the practical application of RT in terms of adaptation and risk potential in healthy and diseased humans.

The aim of this randomized crossover study was, therefore, to investigate hemodynamic cardiopulmonary responses (stroke volume, cardiac output, cardiac work, end-expiratory gas concentrations) during and after strength training with different intensities (50%, 62.5%, and 75% of the 3-Repetition Maximum; 3-RM). Based on the known long-term effects of exercise training, we expect the strongest cardiopulmonary and vascular peripheral responses at 75% of the maximum load.

## Materials and methods

### Participants

Our study was conducted in accordance with the latest version of the Declaration of Helsinki and approved by the Ethics Committee of the Medical Faculty of the University of Leipzig (272/21-ek). Written informed consent was obtained from all participants. The study group consisted of 14 healthy and strength training experienced men (Table 1). Exclusion criteria were cardiac, pulmonary, or inflammatory diseases, athletic inactivity, and orthopedic anomalies at the time of the examinations. In addition, participants had to be able to perform the strength exercises technically and conditionally. We calculated sample size (G*Power software; Heinrich-Heine-Universität Düsseldorf, Germany) according to the expected effect of different load intensities on stroke volume. In our preliminary study, stroke volume rose by 10 ± 16 ml (effect size d = 1.2) at 75% of 3-RM compared to 50% 3-RM. Based on a power of 0.8 with a two-sided paired test and an alpha of 0.05, a sample containing at least 12 participants was required.

**Table 1:** Baseline characteristics of the study participants (n = 14).

Age and performance parameters	
Age (years)	24.5 ± 2.9
Sports Activity (hrs per week)	7.1 ± 2.7
VO_2_max/BM in the IET (ml/min/kg)	44.1 ± 6.2
Three repetition maximum test (3RM kg)	95.1 ± 24.4
Anthropometric parameters	
Height (cm)	183 ± 6.1
Mass (kg)	80.9 ± 7.2
BMI (kg/m^2^)	24.1 ± 2.0
LBM (kg)	67.4 ± 5.6
FM (%)	13.8 ± 3.1
Baseline parameters before IET	
SBP (mmHg)	126.3 ± 8.8
DBP (mmHg)	74.3 ± 7.6
HR (bpm)	77.3 ± 14
SV (ml)	107 ± 13
CO (l/min)	8.2 ± 1.3
CW (J)	1.5 ± 0.2
EDV (ml)	177 ± 23
V_E_ (l/min)	13.6 ± 2.8
VO_2_ (ml/min)	421.5 ± 80

Values are presented as the means and standard deviation; *BM* body mass, *BMI* body mass index, *LBM* lean body mass, *FM* fat mass, *IET* incremental exertion test, *SBP* systolic blood pressure, *DBP* diastolic blood pressure, *HR* heart rate, *SV* stroke volume, *CO* cardiac output, *V*_*E*_ ventilation, *VO*_*2*_ oxygen uptake, *CW* cardiac work, *EDV* end diastolic volume.

### Study design

After the initial pre-examination, all participants completed three experimental sessions involving standardized squats on a smith machine (Technogym Germany GmbH, Germany) over a three-week period. To standardize the training sessions, the individual three-repetition-maximum at 50%, 62.5% and 75% were used. The pre-examination included medical history, lifestyle questionnaire (physical activity, smoking, and alcohol consumption), incremental exercise test (IET), and bioelectrical impedance analysis (Bioimpedance Analyzer BIACORPUS RX 4004 M, MEDI CAL HealthCare GmbH, Germany). The participants performed an incremental exercise test (IET) to exhaustion to determine maximum power output (Pmax), cardiac and pulmonary maximum values.

All participants performed three load conditions on separate days at the same time with a break lasting at least 5 days to ensure adequate recovery. On the first strength training session, the individual 3-repetition maximum (3-RM) after a warm-up period was determined via the approximation method, which corresponds to the maximum amount of weight lifted in clean execution for three repetitions in the smith machine. The 3-RM tests followed the ACSM guidelines for 1-RM tests^31^. After 3-RM determination (mean: 95.1 ± 24.4 kg), participants were allowed to rest for 20 min to reduce potential fatigue effects. Thereafter, they engaged in the training session with 50% of 3-RM load. The training session was then repeated at 62.5% 3-RM and 75% 3-RM loads on separate days in random order. At an additional session, blood pressure was measured during single-armed squatting at 62.5% 3-RM applying the Riva-Rocci/Korotkoff method.

#### Strength training: squats in a smith machine with 50% 3-RM, 62.5% 3-RM, and 75% 3-RM

**Figure 1:**
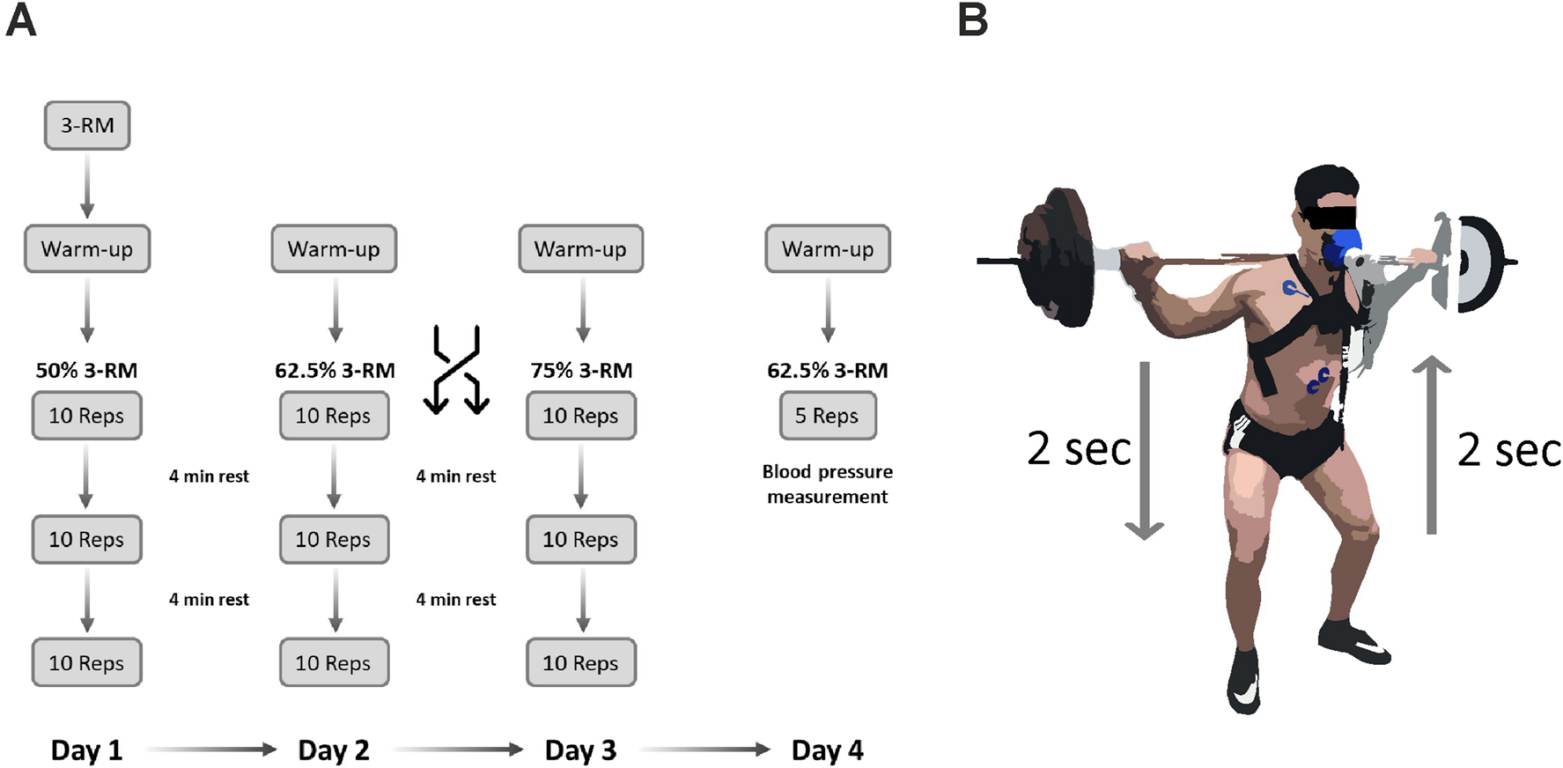
shows the timeline of measurements (**A**) execution and equipment (**B**). (**A**) The three load conditions (3 sets of 10 repetitions interspaced by a resting period of 4 min; intensities: 50%, 62.5% and 75% of 3-RM) were performed on separate days with a break lasting at least 5 days to ensure adequate recovery. The order of sessions 2 and 3 was randomized. On the first day, the individual 3-repetition maximum (3 RM) in the smith-machine back squat was determined. (**B**) Resting and post-exercise period = upright in a smith machine; exercise periods = ten repetitions within 60 s; Execution = 2 s descend, 2 s ascend and 2 s hold at the top; Ventilation and hemodynamic were recorded continuously; blood pressure measurement (only in post-exercise period): immediately after exercise, 1:30 min after exercise, and 2:30 after exercise; Blood pressure measurements during strength training sessions were not taken during the standardized strength training sessions because of interference in the repetition sequence and for safety reasons.

All participants took part in a warm up-period lasting 5 min on a bicycle ergometer (100 W; 75 rpm) at each training session, followed by one set of 10 repetitions without external load, five repetitions with 50% of the subsequent testing load and 3 repetitions with 75% of the subsequent load. For each training session three sets of 10 repetitions were completed for each load with a 4-min rest period between each set^32,33^ to ensure cardiopulmonary and metabolic recovery and to analyze the whole post-exercise period (Fig. 1). Squats were standardized via an individual knee flexion angle and with a timed execution target (2 s descending, 2 s ascending and 2 s hold at the top; i.e. six seconds per repetition). Each subject received visual and auditory feedback regarding correct task execution. Subjects were instructed to avoid any exercise training for 48 h before the sessions.

#### Incremental exertion test (IET)

The IET was performed on a semi-recumbent ergometer (ergometrics 900, ergoline GmbH, Bitz, Germany) at a constant speed of 60–70 rpm. The test started with a 50 W load, which was increased by 15 W every minute until exhaustion occurred. The criteria for exhaustion were a cycling cadence below 60 revolutions per minute, a respiratory quotient above 1.1 and/or reaching the limit of perceived exertion. Each subject continued the test for an additional 5-min recovery period at a 25% load of Pmax. Spirometry (K4b, Cosmed, Italy), thoracic impedance (PhysioFlow, Manatec Biomedical, France), and an electrocardiogram (custo, BT300 custo GmbH, Germany) were synchronized and ran simultaneously during the entire time. Impedance cardiography employs disposable sensors on the neck and chest to measure electrical and impedance changes in the thorax. The change in impedance signal and its timing due to blood flow in the aorta are used to calculate hemodynamic parameters. Furthermore, blood pressure (BP) and rating of perceived exertion (RPE) were recorded every 3 min during the IET. The maximal load and cardiopulmonary parameters (IET 100%) at this level represent the reference for the maximum dynamic workload (Tables 2, 3).

**Table 2:** Mean values during exercise period (n = 14; three sets of 10 repetitions, excluding warm-up and recovery phases).

	50% 3-RM	62,5% 3-RM	75% 3-RM	Effect size η^2^_p_	p-value	IET 100%
Hemodynamic parameters						
SBP (mmHg)	–	197 ± 22.4#	–			
DBP (mmHg)	–	108.8 ± 13.4#	–			
HR (bpm)	128.6 ± 18§	132.5 ± 15§	143.3 ± 16†*	0.54	**< 0.01**	178.9 ± 10
SV (ml)	105.7 ± 14	108.1 ± 18	116.1 ± 20	0.18	0.08	162.4 ± 30
CO (l/min)	13.6 ± 2.4§	14.3 ± 2.5§	16.7 ± 3.7†*	0.56	**< 0.01**	28.9 ± 4.5
EDV (ml)	164.3 ± 25	169.0 ± 32	172.9 ± 30	0.05	0.49	229.8 ± 40
EF (%)	64.9 ± 5.2	64.2 ± 5.7§	67.3 ± 6.3†	0.22	**0.04**	71.1 ± 8.7
Pulmonary parameters						
V_E_ (l/min)	37.6 ± 7.7§	39.6 ± 10.4§	44.0 ± 8.0†*	0.56	**< 0.01**	133.0 ± 26
RR (bpm)	21.1 ± 4.7	20.9 ± 6.6	23.0 ± 5.2	0.13	0.16	42.2 ± 8.4
VT (l)	1.9 ± 0.5	2.0 ± 0.4	2.0 ± 0.4	0.07	0.41	3.2 ± 0.5
VO_2_ (ml/min)	1409 ± 177	1434 ± 239	1530 ± 237	0.16	0.11	3559 ± 428
VO_2_ (ml/(min kg))	17.5 ± 2.7	17.8 ± 3.4	19.0 ± 3.4	0.17	0.09	44.1 ± 6.2
VCO_2_ (ml/min)	1256 ± 180§	13,112 ± 241§	1440 ± 214†*	0.45	**< 0.01**	4036 ± 444
PetO_2_ (mmHg)	103.7 ± 2.6§	105.6 ± 4.9§	108.7 ± 4.5*†	0.54	**< 0.01**	110.7 ± 6.6
PetCO_2_ (mmHg)	38.8 ± 1.6§	38.4 ± 2.7	37.9 ± 2.32*	0.13	0.18	38.3 ± 5.2
avDO_2_ (ml/dl)	10.8 ± 3.0	10.4 ± 2.9	9.6 ± 2.5	0.14	0.14	12.6 ± 2.2
RPE (1–10)	5.1 ± 1.2 §	5.8 ± 1.3§	7.6 ± 0.9*†	0.88	**< 0.01**	9.9 ± 0.3

Significant values are in bold.

Values are presented as the means and standard deviation; *IET* incremental exertion test (the displayed IET data were not included in the ANOVA and only for illustration), *η*^*2*^_*p*_ partial eta-squared of the one way repeated measures ANOVA (50% intensity, 62.5% intensity, 75% intensity), *SBP* systolic blood pressure, *DBP* diastolic blood pressure, *HR* heart rate, *SV* stroke volume, *CO* cardiac output, *EDV* end-diastolic volume, *EF* ejection fraction, *V*_*E*_ ventilation, *RR* respiratory rate, *VT* tidal volume, *VO*_*2*_ oxygen uptake, *VCO*_*2*_ carbon dioxide output, *PetO*_*2*_ end-tidal oxygen-partial pressure, *PetCO*_*2*_ end-tidal carbon dioxide partial pressure, *avDO*_*2*_ arteriovenous difference, *RPE* rating of perceived exertion. * (P < 0.05) different from 50% intensity; † (P < 0.05) different from 62.5% intensity; § (P < 0.05) different from 75% intensity; # = (n = 12) separate measurement with 62.5% of 3-RM.

**Table 3:** Mean values during the post-exercise period (n = 14; mean at one minute after exercise periods).

	50% 3RM	62,5% 3RM	75% 3RM	Effect size η2p	p-value	IET 100%
Hemodynamic parameters						
SBP (mmHg)	135 ± 11	138 ± 11	136 ± 10	0.08	0.34	209 ± 11
DBP (mmHg)	80.4 ± 5.4	80.4 ± 5.5	80.1 ± 5.2	< 0.01	0.90	77.1 ± 8.3
HR (bpm)	126.1 ± 21§	130.6 ± 17§	139.5 ± 17*†	0.47	**< 0.01**	178.9 ± 10
SV (ml)	123.4 ± 17	126.1 ± 22	133.8 ± 26	0.11	0.23	162.4 ± 30
CO (l/min)	15.8 ± 3.8§	16.4 ± .2.4§	18.7 ± 4.3*†	0.39	**< 0.01**	28.9 ± 4.5
EDV (ml)	179.3 ± 26	186.5 ± 30	190.6 ± 36	0.07	0.42	229.8 ± 40
EF (%)	69.0 ± 6.0	67.7 ± 6.1	70.4 ± 7.1	0.13	0.16	71.1 ± 8.7
CW (J)	1.7 ± 0.3	1.8 ± 0.3	1.9 ± 0.4	0.13	0.17	2.9 ± 0.6
Pulmonary parameters						
V_E_ (l/min)	44.6 ± 7.3§†	49.6 ± 7.8§*	58.7 ± 10.7*†	0.83	**< 0.01**	133.0 ± 26
RR (bpm)	20.7 ± 2.9§	21.7 ± 3.9§	24.7 ± 4.4*†	0.53	**< 0.01**	42.2 ± 8.4
VT (l)	2.2 ± 0.3§	2.4 ± 0.4	2.4 ± 0.4*	0.26	**0.02**	3.2 ± 0.5
VO_2_ (ml/min)	1610 ± 183§†	1753 ± 145*	1882 ± 208*	0.48	**< 0.01**	3559 ± 428
VCO_2_ (ml/min)	1491 ± 198§†	1660 ± 169§*	1881 ± 217*†	0.75	**< 0.1**	4036 ± 444
PetO_2_ (mmHg)	103.8 ± 4.6§	105.3 ± 6.4§	109.6 ± 5.3*†	0.63	**< 0.01**	110.7 ± 6.6
PetCO_2_ (mmHg)	40.1 ± 3.1§	39.8 ± 3.3	38.7 ± 3.2*	0.27	**0.02**	38.3 ± 5.2
avDO_2_ (ml/dl)	10.7 ± 2.3	10.9 ± 1.7	10.5 ± 2.6	0.02	0.80	12.6 ± 2.2
TPR (mmHg)	7.0 ± 1.4§	6.6 ± 1.1§	5.9 ± 1.1*†	0.36	**< 0.01**	4.8 ± 0.7

Significant values are in bold.

Values are presented as the means and standard deviation; *IET* incremental exertion test (the displayed IET data were not included in the ANOVA and only for illustration), *η2p* partial eta-squared of the one way repeated measures ANOVA (50% weight loads, 62.5% weight loads, 75% weight loads), *SBP* systolic blood pressure, *DBP* diastolic blood pressure, *HR* heart rate, *SV* stroke volume, *CO* cardiac output, *EDV* end-diastolic volume, *EF* ejection fraction, *CW* cardiac work, *V*_*E*_ ventilation, *RR* respiratory rate, *VT* tidal volume, *VO*_*2*_ oxygen uptake, *VCO*_*2*_ carbon dioxide output, *PetO*_*2*_ end-tidal oxygen partial pressure, *PetCO*_*2*_ end-tidal carbon dioxide partial pressure, *avDO*_*2*_ arteriovenous difference of oxygen, *TPR* total periphery resistance, * (P < 0.05) different from 50% intensity; † (P < 0.05) different from 62.5% intensity; § (P < 0.05) different from 75% intensity.

### Measurements during strength training

The three strength training sessions in this study focused on mean exercise (three sets lasting 1 min), mean immediate post-exercise (1 min after three sets), and cumulated values (three sets with complete post-exercise period). Peak exercise and mean post-exercise values (4 min after three sets) are shown in the supplementary material.

Cardiac output (CO), stroke volume (SV), end-diastolic volume (EDV), ejections fraction (EF), and heart rate (HR) measured by impedance cardiography (sampling interval 10 s), maximum oxygen consumption (VO_2_max), end-tidal oxygen- and carbon dioxide-partial pressure (PetO_2_; PetCO_2_), respiratory rate (RR), tidal volume (VT) and minute ventilation (V_E_) measured by mobile spiroergometry and were monitored continuously at rest, during training, and after the training sessions. For impedance cardiography, six disposable sensors on the neck and chest are used to transmit and detect electrical and impedance changes in the thorax induced by the cardiac flow. The electrodes were applied in a standardized manner after skin preparation (peeling and disinfection) according to the manufacturer's instructions for each training session.

For editing purposes, the values were averaged at 10-s intervals. We calculated mean and peak values during the sets (60 s. excluding warm-up, rest and cool down), as well as mean and peak values during the resting phase (240 s.). For the comparison of the acute post-exercise period (Table 3), the mean value of the first 60 s. after each set was analyzed. We also collected heartbeats, CO, respiration cycles, V_E_, VO_2,_ and VCO_2_ data to compare absolute values during the entire training sessions, including resting and exercise periods.

The arteriovenous oxygen difference (avDO_2_) was calculated using Fick's principle with avDO_2_ = oxygen uptake (V̇O_2_)/cardiac output (CO). Stroke work (SW) was measured in Joules (J) and calculated according to the formula SW = SV × MAP/7.5^34^. Total peripheral resistance (TPR) was determined from heart rate corrected calculated mean arterial blood pressure (MAP = 1/3 × SBP + 2/3 × DBP) × (1 + (HR-60)/1000)^35^ and cardiac output (TPR = MAP/CO).

### Blood pressure assessement during strength training

Blood pressure (BP) and rating of perceived exertion (RPE; from 1 to 10, if 10 was total exhaustion) were observed at rest, immediately after each set, and after 1.30 and 2.30 min of recovery. On a separate day, participants had to perform the squats in a single-arm position in an additional examination to enable blood pressure to be measured while exercising at 62.5% of the 3-RM. An experienced investigator took the blood pressure measurements with an upper arm cuff and a stethoscope. The subject did only one set of 10 repetitions with a slower repetition frequency, and blood pressure was measured indirectly during the eccentric movement between the seventh and tenth repetitions according to the method of Riva-Rocci. We took these measurements in 12 participants of the described study group. Decelerated repetitions of the squats allowed the investigator to move along with them and take the measurements with no interference.

### Statistical analysis

All values are expressed as the means and standard deviation unless otherwise stated, and the significance level was defined as p < 0.05. Data were analyzed using Microsoft Office Excel^®^ 2007 for Windows (Microsoft Corporation, Redmond, Washington, USA) and GraphPad Prism 9 for Windows (GraphPad Software Inc., California, USA). For distribution analysis, the D’Agostino-Pearson normality test was used. If normal distribution was evident, statistical comparisons between the intensity sessions were made using one-way repeated measures ANOVA with Tukey’s post hoc test for multiple comparisons. Otherwise, the nonparametric Friedman test and Dunn post-hoc test were used to compare the different training intensities. Within-group differences were made with a paired Student’s *t*-test.

### Compliance with ethical standards

All procedures described in this study will be performed in accordance with the ethical standards of the responsible committee on human experimentation (institutional and national) and with the principles of the Declaration of Helsinki of 1964 and its latest version. Written informed consent or its equivalent will be obtained from all patients. The protocol has been approved by the Ethics Committee of the Medical Faculty of the University of Leipzig (272/21-ek).

## Results

### Incremental exertion test

Maximum IET values are shown in Table 2. The participants achieved an average maximum power output of 291.1 ± 31.4 W corresponding to relative power of 3.7 ± 0.5 W/kg.

### Cardiopulmonary response during strength training sessions

**Figure 2:**
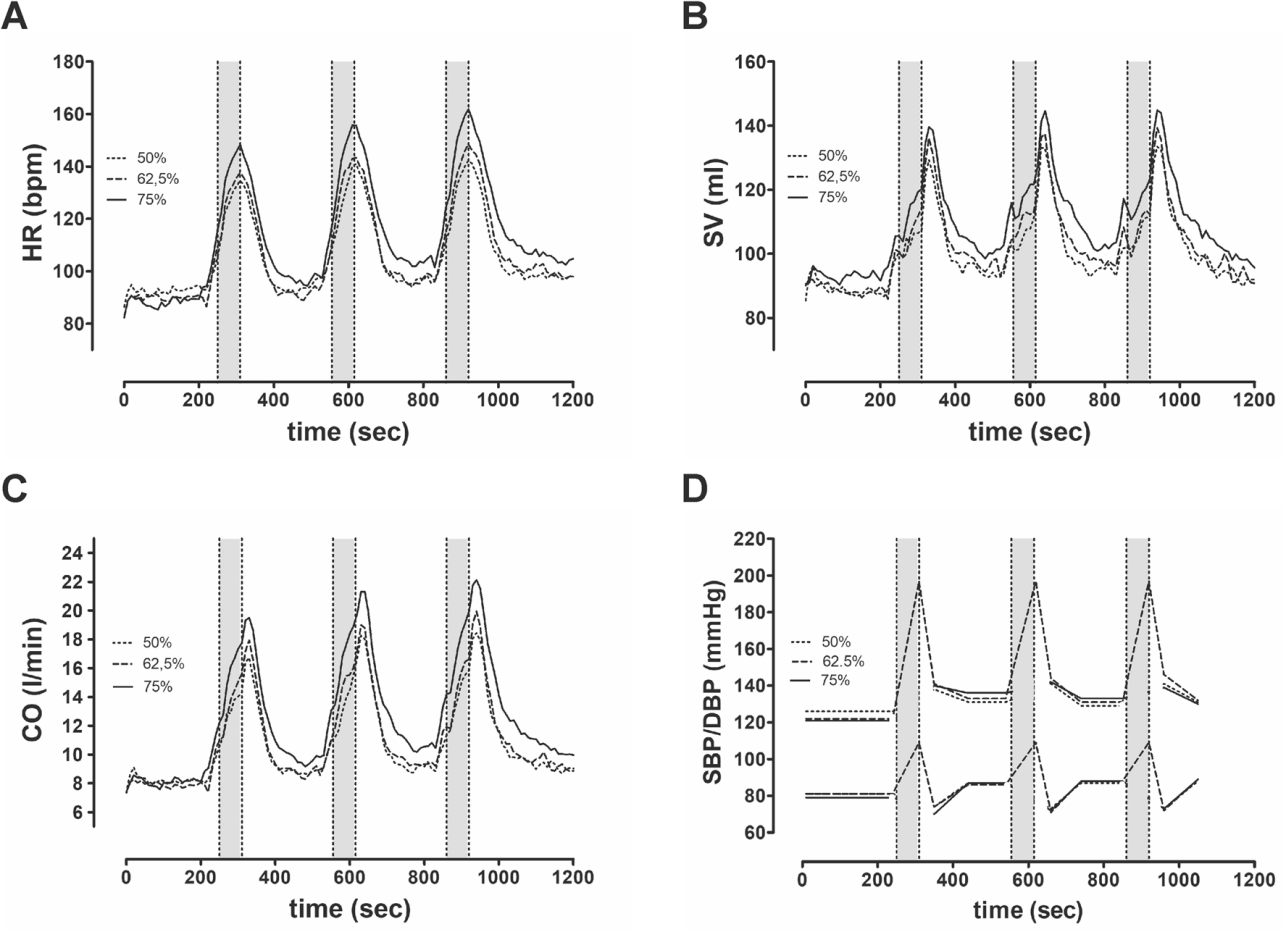
Graphs show the mean cardiac response (n = 14) during strength training sessions with (**A**) heart rate; (**B**) stroke volume, (**C**) cardiac output and (**D**) systolic—and diastolic blood pressure; Exercise blood pressure is only illustrated during 62.5% intensity.

**Figure 3:**
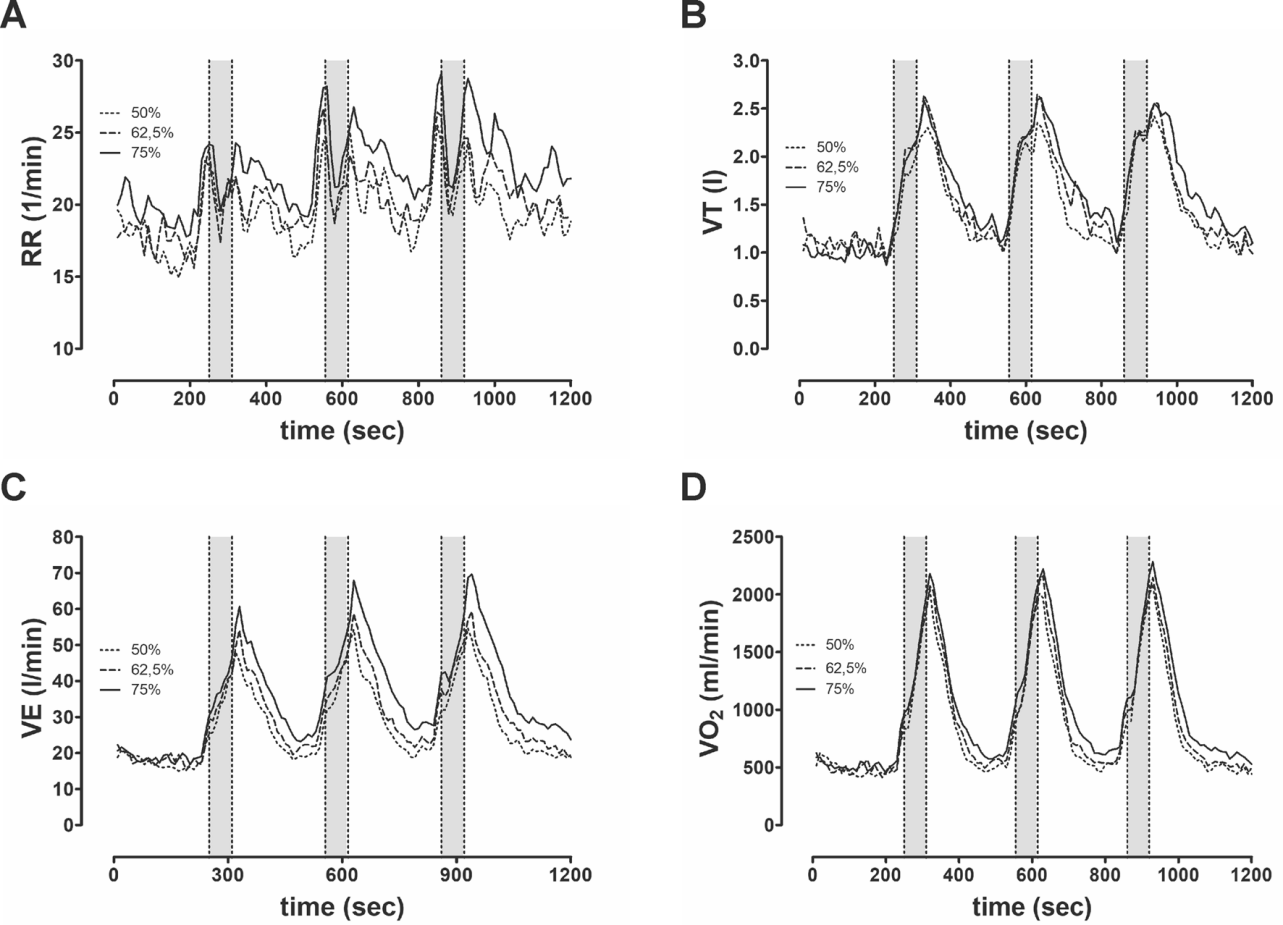
Graphs show the mean pulmonary response (n = 14) during strength training sessions: (**A**) respiratory rate; (**B**) tidal volume, (**C**) minute ventilation and (**D**) oxygen uptake.

**Figure 4:**
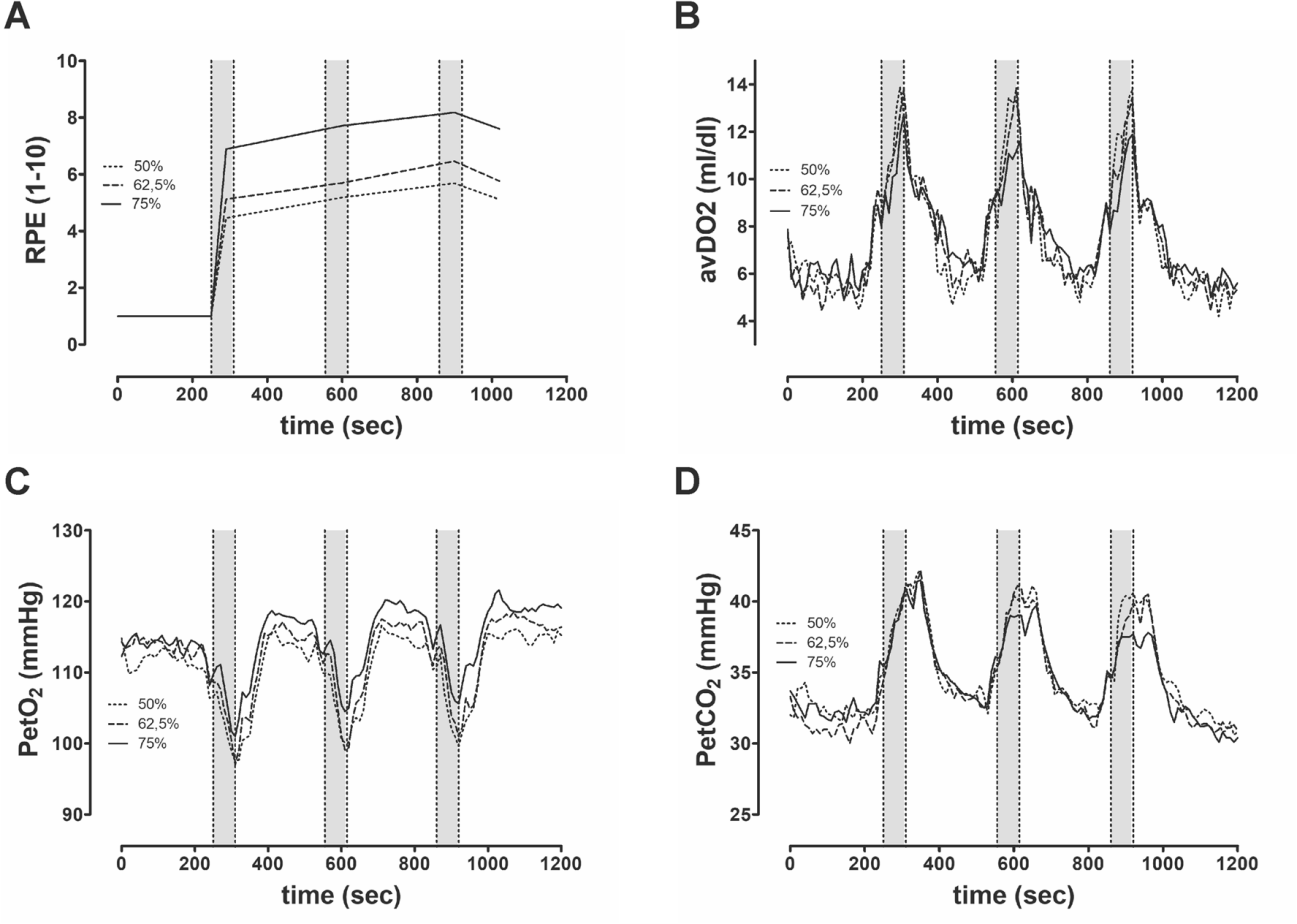
Graphs show the mean periphery and end-tidal course during strength training sessions with (**A**) Rating of perceived exertion; (**B**) Arteriovenous difference of oxygen (**C**) End-tidal oxygen-partial pressure, and (**D**) End-tidal carbon dioxide-partial pressure.

Baseline values were measured prior to each session (values not shown), and we observed no significant hemodynamic differences. Figures 2, 3 and 4 illustrate the time course of cardiac, pulmonary, and periphery responses across the three strength training sessions.

Table 2 shows comparisons of the mean values at the three strength training intensities.

The mean values of the three intensities revealed large differences in the cardiopulmonary response (Table 2). HR and CO showed significantly higher values during 75% intensity than at 50% or at 62.5% of the 3-RM. Ejection fraction (EF%) exhibited significantly higher values during exercise at 75% of the 3-RM than at 62.5% of the 3-RM (67.3 ± 6.3 vs. 64.2 ± 5.7%; p = 0.04). No differences in EF (%) were observed between 50% and 62.5% or 75% of the 3 RMs (64.9 ± 5.2 vs. 64.2 ± 5.7; p = 0.86; 64.9 ± 5.2 vs. 67.3 ± 6.3; p = 0.13).

V_E_ was significantly higher at 75% 3-RM compared to the other two intensities. Our post-hoc comparison yielded significantly lower values in the PetCO_2_ parameter at 75% 3-RM than at 50% 3-RM (37.9 ± 2.32 vs. 38.8 ± 1.6; p = 0.04). PRE was significantly higher at 75% of the 3-RM than at 50% and 62.5% of the 3-RM.

### Cardiopulmonary response immediately after training sessions

Table 3 illustrates significant differences in cardiac (HR and CO) and pulmonary (VT, RR, VO_2_, and VCO_2_) parameters after the strength training (mean of one minute after three sets). Calculated avDO_2_ was the same at all intensities.

### Cumulative values during exercise and resting periods

To compare the three training intensities, the HR, CO, V_E_, VO_2,_ and VCO_2_ parameters were accumulated throughout the training duration (Table 4). Compared with 50% and 62.5%, 75% intensity revealed significantly higher O_2_ consumption, carbon dioxide production, cardiac output, breathing volume, and more breathing cycles as well as heartbeats.

**Table 4:** Cumulated values during exercise and resting periods (three sets of 10 repetitions squats) and recovery periods (three × 4 min after exercise) (n = 14).

	50% of 3-RM	62.5% of 3-RM	75% of 3-RM	Effect size η^2^_p_	p-value
HR (bpm)	1666 ± 288§	1698 ± 263§	1836 ± 253*†	0.08	**< 0.01**
CO (l)	175.4 ± 35§	182.1 ± 29§	211.2 ± 47*†	0.15	**< 0.01**
RR (breaths)	298 ± 43§	309 ± 59§	344 ± 52*†	0.13	**< 0.01**
V_E_ (l)	452.9 ± 80†§	505.2 ± 99*§	602.0 ± 114*†	0.30	**< 0.01**
VO_2_ (l)	14.2 ± 1.9§	15.3 ± 1.9§	17.0 ± 2.2*†	0.25	**< 0.01**
VCO_2_ (l)	13.6 ± 2.1†§	15.2 ± 2.2*§	17.7 ± 2.7*†	0.36	**< 0.01**

Significant values are in bold.

Values are presented as the means and standard deviation; *3-RM* three repetition maximum, *η*^*2*^_*p*_ partial eta-squared of the one-way repeated measures ANOVA, *HR* heart rate, *CO* cardiac output, *RR* respiratory rate, *V*_*E*_ ventilation, *VO*_*2*_ oxygen uptake, *VCO*_*2*_ carbon dioxide output; * (P < 0.05) different from 50% intensity; † (P < 0.05) different from 62.5% intensity; § (P < 0.05) different from 75% intensity.

Figure 2 shows mean responses for HR, CO, SV, and blood pressure time courses across the three strength training intensities. The intensities differed strongly in terms of the cardiopulmonary response with the exception of SV (Fig. 2 and Table 2). Figure 2 also shows that SV increases immediately after the exercise period. Exercise blood pressure is only illustrated during 62.5% of 3-RM intensity.

The course of ventilation parameters (RR; VT; V_E_ and VO_2_) is shown in Fig. 3. We observed significant differences only in the V_E_ value between intensities. RR tended to drop during squat execution and rise after finishing the exercise.

The RPE, calculated avDO_2,_ and the end-tidal gas concentrations of O_2_ and CO_2_ are shown in Fig. 4. RPE during the intensities differed significantly, whereas the avDO_2_ course reveals no differences between intensities. Observing the end-tidal gas concentrations’ course, a substantial change in the exercise period becomes apparent. Normalization does not occur until the post-stress period (Fig. 4). We calculated differences between the exercise and post-exercise periods to illustrate the change in cardiopulmonary parameters (Table 5). SV and CO increased significantly during the post-exercise period compared to the exercise period. Pulmonary parameters (V_E_, VO_2_, VCO_2_, PetCO_2_) increased significantly after the end of exercise compared to the mean exercise-period values, as did the cardiac parameters.

**Table 5:** Change in parameters from exercise period (three sets of 10 repetitions) to post-exercise period (one minute after exercise).

	50% of 3-RM	62.5% of 3-RM	75% of 3-RM	Effect size η^2^_p_	p-value
Hemodynamic parameters					
HR (bpm)	− 2.6 ± 10 p = 0.38	− 2.1 ± 9 p = 0.378	− 3.9 ± 9 p = 0.12	0.048	0.53
SV (ml)	17.6 ± 10 **p = 0.0001**	17.9 ± 14 **p = 0.0004**	17.7 ± 10 **p = 0.0001**	0.0034	0.99
CO (l/min)	2.1 ± 2.5 **p = 0.007**	2.1 ± 1.9 **p = 0.0012**	2.0 ± 1.6 **p = 0.0004**	0.002	0.97
Pulmonary parameters					
V_E_ (l/min)	6.9 ± 5.5§ **p = 0.0004**	9.9 ± 7.7§ **p = 0.0003**	14.7 ± 11*† **p = 0.0002**	0.47	**< 0.01**
VO_2_ (ml/min)	201 ± 204§ **p = 0.0027**	319 ± 236 **p = 0.0002**	353 ± 211* **p = 0.0001**	0.25	**0.02**
VCO_2_ (ml/min)	234 ± 176†§ **p = 0.0003**	348 ± 226* **p = 0.0001**	441 ± 197* **p = 0.0001**	0.449	**< 0.01**
PetO_2_ (mmHg)	− 0.1 ± 3.0 p = 0.89	− 0.29 ± 3.2 p = 0.74	0.87 ± 3.4 p = 0.36	0.098	0.26
PetCO_2_ (mmHg)	1.3 ± 1.7 **p = 0.012**	1.4 ± 1.9 **p = 0.016**	0.8 ± 1.9 **p = 0.15**	0.10	0.25

Significant values are in bold.

Values are presented the difference between post-exercise period and exercise period as the means and standard deviation. The p value in cells represents the comparison between exercise and post-exercise period. *3-RM* three repetition maximum, *HR* heart rate, *SV* stroke volume, *CO* cardiac output, *V*_*E*_ ventilation, *VO*_*2*_ oxygen uptake, *VCO*_*2*_ carbon dioxide output, *PetO*_*2*_ end-tidal oxygen partial pressure, *PetCO*_*2*_ end-tidal carbon dioxide partial pressure; * (P < 0.05) different from 50% intensity; † (P < 0.05) different from 62.5% intensity; § (P < 0.05) different from 75% intensity.

Additional results, such as the peak values during the exercise periods and mean values of the entire post-exercise period are available in the supplement (Supplementary Tables 1 and 2).

## Discussion

The main finding of this randomized cross-over study was the specific post-exercise responses of cardiopulmonary parameters (SV, V_E_, VO_2_, PetO_2_, PetCO_2_). Due to the repetition-dependent breathing pattern with pressurized breathing and a rise in blood pressure during the exercise period, post-exercise cardiopulmonary responses revealed hyperventilation and an increase in stroke volume. As expected, an intensity-dependent cardiopulmonary (HR, CO, EF) response was evident during the exercise period. In contrast, other parameters revealed no intensity-dependent change (VO_2_, VT, RR, SV, EDV) during exercise.

### Pulmonary response

During the exercise period, only V_E_, PetO_2_ and VCO_2_ differed between intensities. However, compared to the IET, V_E_ values during the exercise period remained well below, as expected. Repetition-dependent breathing during the execution period seems to be a causal factor here. General recommendations suggest exhaling during the concentric phase of strength training and inhaling during the eccentric phase^36^. Similarly, in the present study, we specified the execution rhythm—a factor that strongly influenced the respiration pattern with additional pressurized breathing in the “time under tension” (Fig. 3). MacDougall et al. showed that the use of the Valsalva maneuver (VMs) during strength exercises is a natural reflex triggered during resistance exercises when greater effort is required, i.e. increasing proportionally to the work intensity^30,37^. Figure 3 shows the repetition-dependent breathing pattern during exercise. The RR decreases during strength exercise repetitions in all three conditions, and increases again after the load. According to the ventilation values, the PetO_2_ revealed highest values at 75% intensity of 3-RM. Hackett et al. compared strength training with five sets to strength training with two sets and showed that V_E_ was higher and PetCO_2_ lower when the number of sets was higher^38^. Following Hackett et al., both PetO_2_ and PetCO_2_ parameters indicate comparatively higher alveolar ventilation at 75% of 3RM compared to the other intensities^38^. We hypothesized that 75% intensity compared to 62.5%, as well as 50% of the 3-RM, would lead to higher central command and peripheral afferent feedback (in group III/IV) in agreement with the significantly higher RPE and thus explain the higher V_E_ during the higher workload^39–42^. In summary, the respiratory parameters show no differences due to the strength training-related repetition, with the exception of V_E_ and VCO_2,_ which reflect intensity-dependent effort.

During the post-exercise period, all pulmonary parameters exhibited significant intensity-dependent differences. Breathing regulation after the exercise period seems to be affected by the exercise-related breathing pattern. Therefore, the training-induced oxygen deficit and CO_2_ enrichment in blood were more pronounced during higher intensities. As a result, excessive post-exercise oxygen consumption (EPOC) is significantly higher at high intensities (75%) than at medium intensities (62.5%) and this in turn is higher than at low intensities (50%)^43^. This relationship was also evident in the V_E_ characteristics (Table 3). The strong increase in pulmonary parameters during the post-exercise period further indicates execution-induced breath holding during high-intensity strength training. End-expiratory gas concentrations normalized after 1–2 min, thus explaining the afterload hyperventilation^44^.

In summary, an intermittent Valsalva maneuver during exercise has been observed at all three intensities^30,36,37^. Most of our participants’ pulmonary parameters, therefore, exhibited no intensity-dependent differences. Only the pulmonary response in V_E_ was significantly stronger at high intensities (75%) than at low intensities (62.5% and 50%)^14,38^, which was particularly evident during the post-exercise period at all three intensities^38,43^. Nevertheless, during the entire training period, the cumulative parameters between intensities (Table 4) demonstrate a clear intensity-dependent difference, while correspondingly different degrees of the respiratory muscles’ long-term adaptation likely.

### Cardiovascular response

During the exercise period a significant intensity-related increase in HR and CO was evident (Tables 2 and 3; Fig. 2). SV tends to demonstrate an intensity-dependent increase (Tables 2 and 3). We hypothesize that pressurized breathing and increased TPR^37,45^ limit the rise in stroke volume during exercise and thus, depending on its intensity, both increased cardiac inotropy and chronotropy are responsible for elevated CO^46^. Filling pressures (EDV) did not significantly differ between intensities in our study, so that the observed increase in contractility (EF) at 75% intensity is not related to differences in EDV. Rather, increased contractility at 75% intensity appears to be the potential result of a rising heart rate (Bowditch "staircase") and or increased cardiac drive^47,48^.

The present results show that the HR and CO during exercise and during cumulative exercise and resting times was significantly higher at 75% intensity than at the two lower intensities (Table 4). Despite a significantly higher HR during exercise, EDV did not fall at 75% intensity. Thus, the significantly higher EF, as well as the tendency toward increased SV during 75% 3-RM strength training, argues for more afterload-compensating cardiac effort^47^. Therefore, we noticed a pronounced increase in CO induced by the force-frequency relationship (Bowditch effect) during 75% of 3RM, unlike at the other intensities^48^.

We recorded the blood pressure during exercise at 62.5% RM in follow-up examinations. The squats were done with one arm at a slowed repetition frequency. As doing the squats while measuring blood pressure was equivalent to the training sessions except for the lower speed, one can assume the assessed blood pressure values to be representative, with a tendency to higher values^49^. Because of the standardized repetitions (six seconds per repetition) and to ensure the safety of our participants, we were unable to measure the blood pressure during the main examinations. The blood pressure during exercise at 62.5% intensity revealed that both systolic and diastolic values increased substantially during exercise, unlike the resting or post-exercise values. Other investigations have also reported very high strength training-induced blood pressure levels^15,16^. It is well known that an increase in blood pressure depends on the intensity of exercise^15^. There is evidence that cardiac afterload plays a central role in myocardial function during high intensity exercise^46,50^. Figure 2 shows that SV increased only slightly during the exercise period due to the assumed high blood pressure. Once the exercise period is finished, the SV increases abruptly (Fig. 2 and Table 5). This seems to be attributable to the afterload blood pressure. According to Rowland et al.^46^, increasing contractility (EF%) and the heart rate during higher intensity (75% of 3-RM) compared with lower intensity (50% and 62.5% of 3-RM) could be the cardiac response to stronger peripheral resistance during strength exercise^46^. Greater cardiac exercise requirements are reflected in higher heart rates at 75% intensity of 3-RM and explain the higher CO and corresponding VO_2_.

In the post-exercise period SBP and DBP did not differ between intensities. This might relate to decreased vascular resistance (TPR) after strength exercise with higher intensity because of either higher flow-mediated and/or metabolic vasodilation^51^. The consistently significantly higher VCO_2_ values at 75% of 3RM intensity than at the lower loads (50% and 62.5% of 3RM) are a potential explanation for this.

Strength training does not seem to stimulate oxygen uptake substantially more than endurance training^12,13^. The acute cardiac response during strength training is known to be lower than during endurance training^14^, which probably explains the limited long-term effects of strength training on the cardiopulmonary system. However, the strength training in this previous study consisted of body-weight exercises, not equipment-based high intensity-strength training. High-intensity strength training involving equipment will likely elicit stronger cardiopulmonary responses. Aerobic exercise training is known to induce cardiovascular changes, including a functional increase in maximum cardiac output^12^. Cardiac adaptations are thus associated with improved contractility and an increase in blood volume, which raises the SV^52–54^. However, significant improvements in aerobic capacity have also been demonstrated in older adults through high- and low-intensity resistance training^55,56^.

To summarize: the cardiac parameters (unlike the pulmonary parameters due to pressurized breathing), reveal a more distinct intensity-dependent response during exercise. Significant increases in CO are evident during the post-exercise period, which is attributable to the lower blood pressure without load.

### Study limitations

The sample size is small, and we selected only male and recreationally-active participants for this study to prevent compromising interference from possible gender differences in cardiopulmonary function, and muscle performance differences. Therefore, the interpretability and generalizability of the results is limited and only apply to a young male-only and healthy population. The main difficulty during our investigation was measuring blood pressure during strength exercises. For safety reasons (requiring a single-arm execution) and because of the repetition sequence’s influence, we were unable to take blood-pressure measurements during our participants’ strength sessions. We thus cannot quantify differences in blood pressure during exercise. Nevertheless, we did take single-arm blood pressure measurements (Riva-Rocci/Korotkoff) in 12 participants at an additional session during which the squats were performed at a slower repetition frequency (62.5% 3-RM). Cardiac parameters obtained via impedance cardiography may be overestimated using absolute values^57^. However, since intra-individual differences were compared, changes in these parameters were more relevant than the absolute values. Other working groups have carried out thoracic impedance cardiography to detect intra-individual changes in SV and CO^28,58,59^. Stroke volume and cardiac output measurements via impedance cardiography shows acceptable agreement compared with magnet resonance imaging and direct Fick or thermodilution methods^57,60,61^.

## Conclusions

With this randomized cross-over study, we examined the acute hemodynamic response to standardized strength training at different intensities but of the same duration and muscle mass and during exercise and post-exercise periods. The cumulative cardiopulmonary response of exercise and post-exercise periods corresponds to the intensity differences. However, during the exercise period, a repetition-dependent breathing pattern was observed. Equipment-supported high-intensity strength training resulted in repetition-adjusted ventilation with breath holding and a markedly increase in blood pressure during the exercise period. Blood pressure dropped immediately after exercise, followed by a substantial increase in stroke volume during the immediate post-exercise period. The training-induced oxygen deficit and CO_2_ enrichment in blood is compensated by post-exercise hyperventilation. The post-exercise period in strength training thus differs from that in endurance training but is, however, probably associated with proven long-term cardiopulmonary adaptations in strength training. Nevertheless, the results of this study were conducted with healthy, recreationally-active and male individuals and are not generally transferable to other cohorts. In this context, high strength training intensity is of critical importance concerning specific cardiopulmonary adaptations, while pressurized breathing should be avoided and only moderate intensity should be used during rehabilitation.

## Supplementary Information

Supplementary Information.

## Data Availability

The original contributions presented in the study are included in the article’s supplementary material; further inquiries can be directed to the corresponding author/s.

## Abbreviations

avDO_2_: Arteriovenous oxygen content difference
CO: Cardiac output
CW: Cardiac work
DBP: Diastolic blood pressure
EDV: End diastolic volume
EF%: Ejection fraction
HR: Heart rate
IET: Incremental exertion test
LAC: Blood lactate concentration
RR: Respiration rate
SBP: Systolic blood pressure
SV: Stroke volume
TPR: Total peripheral resistance
V_E_: Minute ventilation
VCO_2_: Carbon dioxide
VO_2_: Oxygen uptake
