# Supplementary material for: Intensity-dependent cardiopulmonary response during and after strength training
Source: Sci Rep. 2023 Apr 24;13:6632. doi: 10.1038/s41598-023-33873-x (PMC10126007; doi:10.1038/s41598-023-33873-x)
Supplement: Supplementary file 1 — Supplementary Information. [file 41598_2023_33873_MOESM1_ESM.docx]

**Supplement**

**Table 1 Peak values during exercise period (n = 14; excluding warm-up and recovery phases)**

|  | **50 % 3-RM** | **62,5 % 3-RM** | **75 % 3-RM** | **Effect size η2p** | **p-value** |
| --- | --- | --- | --- | --- | --- |
| **Hemodynamic Parameters** |  |  |  |  |  |
| SBP (mmHg) |  | 197±22.4# |  |  |  |
| DBP (mmHg) |  | 108.8±13.4# |  |  |  |
| HR (bpm) | 137.4±18§ | 141.7±16§ | 153.8±16†* | .64 | <.01 |
| SV (ml) | 109.2±17 | 112.8±22 | 121.1±20 | .19 | .07 |
| CO (l/min) | 15.0±2.5§ | 15.9±.3.1§ | 18.6±3.8†* | .61 | <.01 |
| EDV (ml) | 166.6±27 | 173.3±35 | 177.3±31 | .07 | .39 |
| EF (%) | 66.1±5.7 | 65.2±6.1 | 68.5±6.7 | .20 | .06 |
| CW (J) |  | 2.3±0.5# |  |  |  |
| **Pulmonary Parameters** |  |  |  |  |  |
| V_E_ (l/min) | 44.6±8.2§ | 45.1±12.7§ | 50.2±8.7†* | .31 | <.01 |
| RR (bpm) | 22.0±5.5 | 22.1±8.7 | 23.7±5.6 | .05 | .53 |
| VT (l) | 2.1±0.5 | 2.2±0.5 | 2.2±0.4 | <.01 | .92 |
| VO_2_ (ml/min) | 1845±211 | 1837±273 | 1958±269 | .10 | .24 |
| VO_2_ (ml/(min/kg)) | 22.9±3.2 | 22.8±4.0 | 24.3±4.1 | .11 | .22 |
| VCO_2_ (ml/min) | 1547±181§ | 1546±274§ | 1693±252†* | .25 | .03 |
| PetO_2_ (mmHg) | 99.6±3.8§ | 100.1±6.5§ | 104.1±4.6†* | .46 | <.01 |
| PetCO_2_ (mmHg) | 40.3±2.1 | 40.3±3.5 | 39.0±2.4 | .20 | .06 |
|  |  |  |  |  |  |
| avDO_2_ (ml/dl) | 12.8±3.2 | 12.1±3.4 | 11.0±2.7 | .19 | .07 |
| TPR (mmHg) |  | 9.8±2.6# |  |  |  |

Values are presented as the means and standard deviation; η^2^_p_ = partial eta-squared of the one way repeated measures ANOVA (50 % weight loads, 62.5 % weight loads, 75 % weight loads); SBP = systolic blood pressure; DBP = diastolic blood pressure; HR = heart rate; SV = stroke volume; CO = cardiac output; CW = cardiac work; VE = ventilation; RR = respiratory rate, VT = tidal volume, VO_2_ = oxygen uptake; VCO_2_ = carbon dioxide output; PetO_2 =_ end-tidal oxygen partial pressure, PetCO_2_ = end-tidal carbon dioxide partial pressure, avDO_2_ = arteriovenous difference of oxygen, TPR= total periphery resistance,* (P < 0.05) different from 50 % intensity; † (P < 0.05) different from 62.5 % intensity; § (P<0.05) different from 75 % intensity; # = (n=12) separate measurement with 62.5 % of 3-RM.

**Table 2 Mean values during the post-exercise period (n = 14; mean of four minutes after exercise period)**

|  | **50 % 3RM** | **62,5 % 3RM** | **75 % 3RM** | **Effect size η2p** | **p-value** |
| --- | --- | --- | --- | --- | --- |
| **Hemodynamic Parameters** |  |  |  |  |  |
| SBP (mmHg) | 135.4±11 | 137.6±11 | 136.3±10 | .08 | .34 |
| DBP (mmHg) | 80.4±5.4 | 80.4±5.5 | 80.1±5.2 | <.01 | .90 |
| HR (bpm) | 104.8±20§ | 106.7±17§ | 114.3±17*† | .34 | <.01 |
| SV (ml) | 103.6±10 | 106.2±15 | 113.3±22 | .13 | .16 |
| CO (l/min) | 11.1±2.5§ | 11.4±.1.7§ | 13.1±3.0*† | .40 | <.01 |
| EDV (ml) | 159.8±18 | 166.5±24 | 169.6±31 | .07 | .37 |
| EF (%) | 64.9±4.7 | 63.8±5.9 | 66.8±6.1 | .16 | .10 |
| CW (J) | 1.4±0.2 | 1.5±0.2 | 1.6±0.3 | .14 | .14 |
| **Pulmonary Parameters** |  |  |  |  |  |
| V_E_ (l/min) | 28.9±5.2§† | 32.9±6.3§* | 39.5±8.3*† | .80 | <.01 |
| RR (bpm) | 19.8±2.8§ | 20.8±3.7§ | 23.1±3.9*† | .67 | <.01 |
| VT (l) | 1.5±0.3§ | 1.6±0.3 | 1.8±0.4* | .77 | <.01 |
| VO_2_ (ml/min) | 853±135§† | 938±116§* | 1050±158*† | .56 | <.01 |
| VCO_2_ (ml/min) | 842±149§† | 959±152§* | 1132±194*† | .55 | <0.1 |
| PetO_2_ (mmHg) | 111.5±2.2§ | 113.3±4.4§ | 116.0±4.4*† | .49 | <.01 |
| PetCO_2_ (mmHg) | 35.1±2.0 | 34.8±2.2 | 34.6±2.3 | .07 | .40 |
|  |  |  |  |  |  |
| avDO_2_ (ml/dl) | 12.8±3.2 | 12.1±3.4 | 11.0±2.7 | .04 | .57 |
| TPR (mmHg) | 9.7±1.8§ | 9.3±1.5§ | 8.3±1.6*† | .40 | <.01 |

Values are presented as the means and standard deviation; η2p = partial eta-squared of the one way repeated measures ANOVA (50 % weight loads, 62.5 % weight loads, 75 % weight loads); SBP = systolic blood pressure; DBP = diastolic blood pressure; HR = heart rate; SV = stroke volume; CO = cardiac output; CW = cardiac work; VE = ventilation; RR = respiratory rate, VT = tidal volume, VO_2_ = oxygen uptake; VCO_2_ = carbon dioxide output; PetO_2_ = end-tidal oxygen partial pressure, PetCO_2_ = end-tidal carbon dioxide partial pressure, avDO_2_ = arteriovenous difference of oxygen, TPR= total periphery resistance, * (P < 0.05) different from 50 % intensity; † (P < 0.05) different from 62.5 % intensity; § (P<0.05) different from 75 % intensity.
